# MicroRNA signature from extracellular vesicles of HCV/HIV co-infected individuals differs from HCV mono-infected

**DOI:** 10.1007/s00109-023-02367-8

**Published:** 2023-09-14

**Authors:** Victoria Cairoli, Daniel Valle-Millares, María C. Terrón-Orellano, Daniel Luque, Pablo Ryan, Lourdes Dominguez, Luz Martín-Carbonero, Ignacio De los Santos, Elena De Matteo, Beatriz Ameigeiras, Verónica Briz, Paola Casciato, María Victoria Preciado, Pamela Valva, Amanda Fernández-Rodríguez

**Affiliations:** 1 Multidisciplinary Institute for Investigation in Pediatric Pathologies (IMIPP), CONICET-GCBA, Laboratory of Molecular Biology, Pathology Division, Ricardo Gutiérrez Children’s Hospital, C1425EFD CABA Buenos Aires, Argentina; 2 Unit of Viral Infection and Immunity, Centro Nacional de Mirobiología, Instituto de Salud Carlos III (ISCIII), 28222 Majadahonda, Madrid, Spain; 3 Unit of Electron Microscopy Scientific and Technical Central Units (UCCT), Health Institute Carlos III (ISCIII), 28222 Majadahonda, Madrid, Spain; 4 Infectious Diseases Department, Internal Medicine Department HIV/Hepatitis, Infanta Leonor University Hospital, 28031 Madrid, Spain; 5 Centro de Investigación Biomédica en Red de Enfermedades Infecciosas (CIBERINFEC), Instituto de Salud Carlos III, 28222 Madrid, Spain; 6 HIV Unit, Internal Medicine Department, Research Institute of the Hospital, 12 de Octubre (imas12), 28041 Madrid, Spain; 7 Infectious Diseases Unit, Internal Medicine Department, La Paz University Hospital, IdiPAZ, 28046 Madrid, Spain; 8 Infectious Diseases Unit, Internal Medicine Department, La Princesa University Hospital, 28006 Madrid, Spain; 9 Liver Unit, Ramos Mejía Hospital, C1221ADC CABA Buenos Aires, Argentina; 10 Viral Hepatitis Reference and Research Laboratory, Centro Nacional de Microbiología, Instituto de Salud Carlos III, 28222 Majadahonda, Madrid, Spain; 11 Liver Unit, Italian’s Hospital of Buenos Aires, C1199 CABA Buenos Aires, Argentina; 12 Centro Nacional de Microbiología, Instituto de Salud Carlos III, Carretera Majadahonda, Pozuelo, Km 2.2, 28220 Majadahonda, Madrid, Spain

**Keywords:** Chronic hepatitis C infection, HCV/HIV coinfection, miRNAs, Pathogenesis, Liver diseases, Extracellular vesicles

## Abstract

**Abstract:**

Hepatitis C virus (HCV) coinfection with human immunodeficiency virus (HIV) has a detrimental impact on disease progression. Increasing evidence points to extracellular vesicles (EVs) as important players of the host-viral cross-talk. The microRNAs (miRNAs), as essential components of EVs cargo, are key regulators of normal cellular processes and also promote viral replication, viral pathogenesis, and disease progression. We aimed to characterize the plasma-derived EVs miRNA signature of chronic HCV infected and HIV coinfected patients to unravel the molecular mechanisms of coinfection. EVs were purified and characterized from 50 plasma samples (21 HCV mono- and 29 HCV/HIV co-infected). EV-derived small RNAs were isolated and analyzed by massive sequencing. Known and de novo miRNAs were identified with miRDeep2. Significant differentially expressed (SDE) miRNA identification was performed with generalized linear models and their putative dysregulated biological pathways were evaluated. Study groups were similar for most clinical and epidemiological characteristics. No differences were observed in EVs size or concentration between groups. Therefore, HCV/HIV co-infection condition did not affect the concentration or size of EVs but produced a disturbance in plasma-derived EVs miRNA cargo. Thus, a total of 149 miRNAs were identified (143 known and 6 de novo) leading to 37 SDE miRNAs of which 15 were upregulated and 22 downregulated in HCV/HIV co-infected patients. SDE miRNAs regulate genes involved in inflammation, fibrosis, and cancer, modulating different biological pathways related to HCV and HIV pathogenesis. These findings may help to develop new generation biomarkers and treatment strategies, in addition to elucidate the mechanisms underlying virus–host interaction.

**Key messages:**

- HCV and HCV/HIV displayed similar plasma-EV size and concentration.
- EVs- derived miRNA profile was characterized by NGS.
- 37 SDE miRNAs between HCV and HCV/HIV were observed.
- SDE miRNAs regulate genes involved in inflammation, fibrosis and cancer.

**Supplementary Information:**

The online version contains supplementary material available at 10.1007/s00109-023-02367-8.

## Introduction

Hepatitis C virus (HCV) and human immunodeficiency virus (HIV) infections are two persistent public health challenges [1]. HCV causes chronic infection in approximately 70% of the people exposed to the virus. Chronic hepatitis C (CHC) patients often remain asymptomatic until the liver is seriously damaged, which may result in the development of cirrhosis, hepatocellular carcinoma (HCC), and ultimately death [2]. HCV and HIV coinfection is a rather common condition since both viruses share transmission routes [3]. It is estimated that 6.2% of the people living with HIV have serological evidence of HCV exposure [1]. Moreover, those HCV positive individuals who are also infected with HIV show a worse disease outcome, with higher rates of cirrhosis, liver failure, and HCC [4].

Both viruses modulate the immune response and, although the molecular bases of HCV and HIV-induced immune dysfunction are still incompletely understood, increasing evidence points to extracellular vesicles (EVs) as important players of the cellular cross-talk between infected and immune cells [5]. Currently, the term EVs refers to particles naturally released from cells that are delimited by a lipid bilayer without replicating ability. According to their size, EVs can be classified as small (< 200 nm) or medium/large (> 200 nm) [6]. They are released into the extracellular milieu and are found in most biological fluids. EVs carry proteins, nucleic acids including microRNAs (miRNAs), and lipids with a specific composition that characterizes the cell of origin, suggesting an active non-random packaging of its content, that generates a response in the receptor cell [7]. Thus, EVs are involved in intercellular signaling both of innate and adaptive immune response against pathogens, being essential in the pathogenesis of viral hepatitis and its associated liver diseases [8]. Together with HCV, HIV also hijacks the EVs’ machinery to evade immune surveillance, promoting infection and dissemination [9]. Therefore, both viruses modify the EVs’ cargo produced by infected cells, such as miRNAs and proteins, among others [10, 11]. The miRNAs are highly conserved short non-coding RNAs (20–24 nts) fine-tuning regulators of gene expression [12]. Hence, the EVs–miRNA profile is context dependent, providing information about the infectious dynamics and the virus-host relationship [7]. The key regulatory role of miRNAs has particularly emerged in chronic liver diseases; so, a dysregulation of their expression has been well-described in liver tissue and PBMCs, where we found that HCV exposure strongly influences the miRNome in HIV patients, leading to specific miRNA signatures [13, 14]. However, little is known about the EVs–miRNAs regulation under HCV/HIV coinfection.

Currently, the direct-acting antivirals allow rapid elimination of HCV, but this therapy is not globally available, and some patients do not necessarily achieve liver disease cure or, at least, stop its progression. Likewise, antiretroviral therapies (ART) achieve virological suppression in most HIV individuals; however, HIV persistence in cell reservoirs is an ongoing concern, and it has been described that miRNAs are involved in the latency process [15]. Given this scenario, an in-depth understanding of the pathogenesis of CHC and HCV/HIV coinfection will ultimately allow the development of treatment, diagnoses, and monitoring systems for liver diseases that improve the quality of life of infected patients.

Many questions remain unanswered about the characterization of the miRNA fingerprint in different pathological situations and scarce data about miRNA–EVs has been published and none with a massive approach. Thus, our study aims to massively characterize the miRNA profile in purified EVs from plasma of HCV mono-infected and HCV/HIV co-infected patients and to assess the specific miRNA signature of each group of patients, as well as their potential biological function.

## Materials and methods

### Patients

The study population included a total of 50 CHC patients, *n* = 21 HCV mono-infected and *n* = 29 HCV/HIV co-infected. HCV mono-infected patients were recruited from Italian’s Hospital of Buenos Aires and Ramos Mejía Hospital from Buenos Aires, Argentina, and HCV/HIV co-infected patients from La Paz University Hospital, Infanta Leonor University Hospital, La Princesa University Hospital, Puerta de Hierro, and 12 de Octubre Hospital from Madrid, Spain.

Patients were naive of treatment for HCV. CHC infection was defined by the presence of anti-HCV antibodies in serum and detectable HCV RNA in plasma samples in at least 2 separate occasions. HIV diagnosis was assessed by HIV antibodies presence. All HCV/HIV co-infected patients received suppressive antiretroviral treatment (ART) for at least 1 year. They maintained undetectable level of HIV with CD4+ T-cells counts ≥ 500 cells/mm^3^ since at least 1 year before sample collection.

Patients had no other causes of liver disease, autoimmune or metabolic disorders, and HCC or co-infection with hepatitis B virus (HBV). Cases with alcohol consumption (men > 30 g/day; women > 20 g/day) were excluded.

Clinical and epidemiological data were obtained from medical records. Liver fibrosis was assessed at time of blood sample collection by histological observation of liver biopsies according to METAVIR in HCV mono-infected cases and by transient elastography in HCV/HIV co-infected patients [16]. Cases were categorized as significant (≥ 2) and no significant (< 2) fibrosis.

Informed written consent was obtained from each patient. The study protocol conformed to the ethical guidelines of the 1975 Declaration of Helsinki and has the approval of the ethics committees of all institutions.

### Extracellular vesicle isolation

Whole peripheral blood was extracted and processed within the first 4 h after extraction. Plasma fraction was pre-clarified at 648 g 15 min at 4 °C and stored at −80 °C.

EVs’ isolation was performed using the ExoRNeasy Serum/Plasma Midi kit (QIAGEN, cat #77044) according to manufacturer’s instructions with custom modifications. All samples were processed at the National Center for Microbiology (Madrid, Spain). Briefly, 1.2 ml of EDTA-anticoagulated plasma was diluted in pre-filtered phosphate buffered saline (PBS), centrifuged at 2.000 g and 10.000 g consecutively and filtered through a 0.2-µm pore membrane. Samples were then diluted with binding buffer and transferred to a spin column. EVs retained in the membrane were washed and eluted (Buffer XE, QIAGEN, cat #76214). Aliquots for further EV characterization were stored at −80 °C.

### EVs characterization

#### Electron microscopy (EM)

EVs were negatively stained for observation. Briefly, a 5-µl aliquot of EVs was mixed with 5 µl PFA 4%, vortexed and incubated for at least 5 min at room temperature (RT). Vesicles were then adsorbed on glow-discharged collodion carbon grids, washed two times with MilliQ water and stained with 2% uranyl acetate for 1 min. Samples were imaged on a FEI Tecnai 12 electron microscope operated at 120 kV and equipped with a FEI Ceta CCD camera. An average of 20 micrographs per sample was evaluated and EV diameter was measured with FIJI/ImageJ [17]. In addition, cryo-EM analysis was performed from a concentrated sample of EVs (starting material: 7 ml of plasma). Samples were applied to Quantifoil Cu/Rh R2/2 300 mesh glow-discharged grids and vitrified using a Leica EM GP2 cryofixation unit. Data was collected on a Talos cryoelectron microscope (Thermo Fisher Scientific) operated at 200 kV, and images were recorded with Falcon 3 direct electron detector (Thermo Fisher Scientific) in lineal mode using the EPU automated data acquisition software (Thermo Fisher Scientific) for single particle analysis.

#### Nanoparticle tracking analysis (NTA)

Size distribution and concentration of EVs were determined by analyzing the light scattering and the Brownian motion of the suspended particles in pre-filtered PBS using a NanoSight NS300 system (Malvern) equipped with a fast video capture and particle-tracking software (NanoSight NTA 3.4). Briefly, samples were diluted 100-fold using PBS pre-filtered through a 0.2-µm pore membrane filter and analyzed in triplicate for 60 s per replicate at 25 frames per second (fps).

### High throughput sequencing and bioinformatics analysis

Total RNA was purified from eluted EVs with the ExoRNeasy Serum/Plasma Midi kit (QIAGEN, cat #77044). RNA quantity and quality were evaluated by Nanodrop (Thermo Fisher Scientific). Purity and integrity evaluation, small RNA library preparation, and sequencing were performed at the Centre for Genomics Regulation (CRG) (Barcelona, Spain). Size distribution of the RNA was evaluated by Bioanalyzer 2100 with Agilent RNA 6000 pico kit (Agilent). A small RNA library was constructed with NEBNext Multiplex Small RNA Library Prep Kit (NewEngland BioLabs) following the manufacturer’s instructions including a different index for each sample. Sequencing was performed on the Illumina HiSeq2500 platform, single-end-sequencing, 50 nts (1 × 50) to get roughly 10 million reads per sample.

Raw data was analyzed using a specific bioinformatic pipeline for the identification of known and novel miRNAs detailed in Supplementary Data 1. Briefly, reads were quality checked with FastQC, and adapter trimming was performed with cutadapt. Remaining reads were analyzed with miRDeep2 to identify and quantify known and unknown miRNAs.

## Statistical analysis

For the descriptive analysis, significant differences between categorical data were calculated using the chi-squared test and Fisher’s exact test. Student *t*-test and Mann-Whitney *U* test were used to compare parametric and non-parametric continuous variables among independent groups, respectively. The miRNA count matrix was filtered and normalized with Trimmed Mean of M-values (TMM) and count per million (CPM) using edgeR. Significant differentially expressed (SDE) miRNAs between groups (HCV mono-infected vs HCV/HIV co-infected) were analyzed using a generalized linear model with negative binomial distribution (bnGLM) adjusted by liver fibrosis. The miRNAs with fold change (FC) ≥ 1.5 (|log FC| ≥ 0.585) and *q*-value ≤ 0.05 (*p*-value corrected for the false discovery rate (FDR) by Benjamini-Hochberg correction) were considered significant. Specific analysis on miRNA sequencing revealed that a minimum of 19 individuals per group is required to achieve a minimum 1.5-fold change with an average power exceeding 80% [18]; therefore, sample size of the present study is sufficiently large to reliably detect the required 1.5-fold expression differences. See Supplementary Data 1 for extended details.

Statistical software R (v4.0.2) (R Foundation for statistical computing, Vienna, Austria) was used for all statistical analyses.

### miRNA-based target prediction and pathway enrichment analysis

SDE miRNA-target interactions and pathway enrichment analysis of the target genes were performed in silico, as previously described [14]. Only experimentally validated interactions were considered. Extended details are in Supplementary Data 1.

## Results

### Clinical characteristics of each group of patients

Clinical and epidemiological characteristics of HCV mono-infected and HCV/HIV co-infected patients enrolled in this study are summarized in Table 1. Groups were homogeneously balanced according to sex, age, weight, body mass index (BMI), and other clinical characteristics. The HCV mono-infected group showed significantly higher levels of alkaline phosphatase (ALP) (*p* = 0.021). HCV genotype 1 was the most prevalent in HCV mono-infected patients (*p* = 0.024).

**Table 1:** Clinical and epidemiological characteristics of HCV mono-infected and HCV/HIV co-infected patients

**Variables**	**HCV**	**HCV/HIV**	***P*** **value**
***N***	21	29	
**Sex (% female)**	11 (52.4%)	14 (48.3%)	0.407
**Age (years)**	54 (46.5; 62.5)	50 (45; 53)	0.142
**Weight (kg)**	70.1 (60; 81.2)	64.7 (54.7; 76.2)	0.241
**Height (cm)**	170.5 (160; 175.3)	167.5 (161.5; 170.3)	0.849
**BMI (kg/m**^**2**^**)**	26.1 (22; 27.4)	23.1 (20.7; 26.5)	0.238
**Risk of infection**			
** Parenteral**	7 (33.3%)	16 (55.2%)	0.686^a^
** Sexual**	2 (9.5%)	8 (27.6%)	
** Unknown**	12 (57.2%)	5 (17.2%)	
** Time of HIV infection (months)**		268.3 (163.4; 332.6)	
**HCV Genotype**			
** 1**	19 (90.4%)	17 (58.6%)	0.024*
** 2, 3, and 4**	2 (9.6%)	12 (41.4%)	
**HCV viral load (UI/ml)**	2.5E06 (8.8E05; 6.4E06)	4.4E06 (6.6E05; 1.1E07)	0.548
**Fibrosis severity**			
** F < 2**	10 (47.6%)	22 (70.96%)	0.072
** F ≥ 2**	11 (52.4%)	7 (22.58%)	
**TC (mg/dL)**	189 (177; 219)	186 (166; 199.8)	0.714
**GOT (UI/ml)**	60 (41.7; 101)	37 (27.5; 61)	0.071
**GPT (UI/ml)**	67 (43.5; 163.5)	43 (32; 62.5)	0.051
**GGT (UI/ml)**	42 (21.2; 126.5)	65 (33; 118.3)	0.538
**ALP (UI/ml)**	101 (89; 127)	85.5 (63.2; 104.5)	0.020*
**Platelets (10**^**3**^**/ul)**	188.2 (100.7; 223.2)	201 (96.8; 226.2)	0.995
**CD4+ T-cell (cell/mm**^**3**^**)**	735.6 (611.0; 1260)	703.8 (528.0; 1010)	0.243

Values expressed as cases (%) or median (percentile 25; percentile 75)

*HCV* Hepatitis C Virus, *HIV* Human Immunodeficiency Virus, *BMI* Body Mass Index, *TC* total cholesterol, *GOT* Glutamic oxaloacetic transaminase, *GPT* glutamic pyruvic transaminase, *GGT* gamma-glutamyl transferase, *ALP* alkaline phosphatase

^*^Statistically significant differences between HCV mono-infected and HCV/HIV co-infected group (*p* < 0.05)

^a^No significant differences between parenteral and sexual risk of infection

### EV characterization

The isolated EVs’ size and shape were evaluated by TEM and cryo-EM. TEM image analysis revealed the presence of spheric particles with a mean diameter of ~130 nm, ranging from 63 to 230 nm (Supplementary Data 2a). Cryo-EM also showed different sized spherical particles with a lipid bilayer (Supplementary Data 2b). No significant differences in particle size or concentration were observed between HCV mono-infected and HCV/HIV co-infected groups (Supplementary Data 3).

### EV-derived miRNA comparison between study groups

**Fig. 1:**
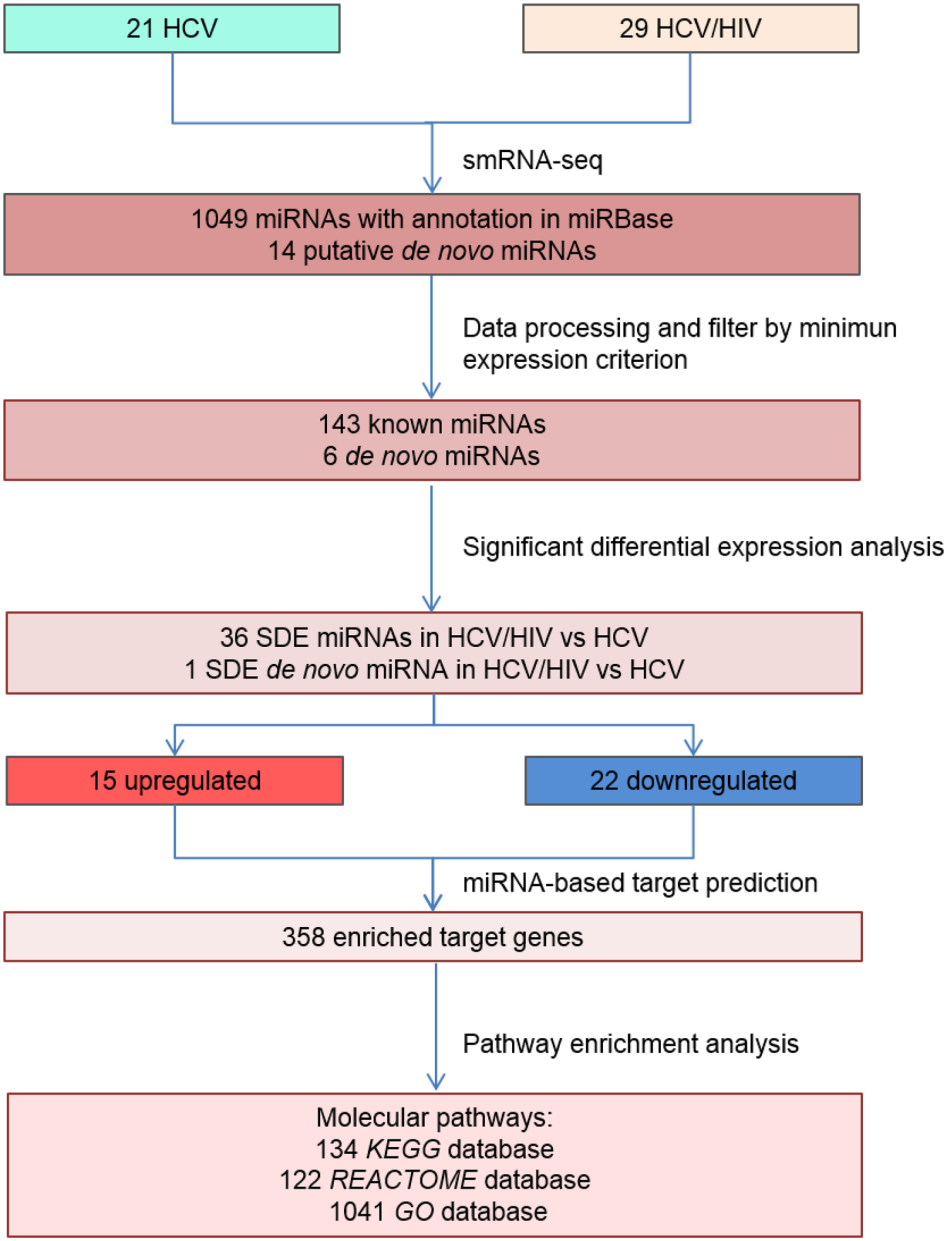
Schematic representation of RNA-seq results analysis. miRNA sequencing analysis and summary of the results

The raw sequencing data has been deposited in the ArrayExpress repository (EMBL-EBI) under accession number E-MTAB-11811. On average, 10 million reads per sample were obtained, which is an appropriate depth for analysis. A total of 1049 known miRNAs were identified, plus 14 putative de novo miRNAs. After filtering (see Supplementary Data 1 for details), 149 miRNAs (143 known and 6 de novo) remained for subsequent analysis (Fig. 1).

**Fig. 2:**
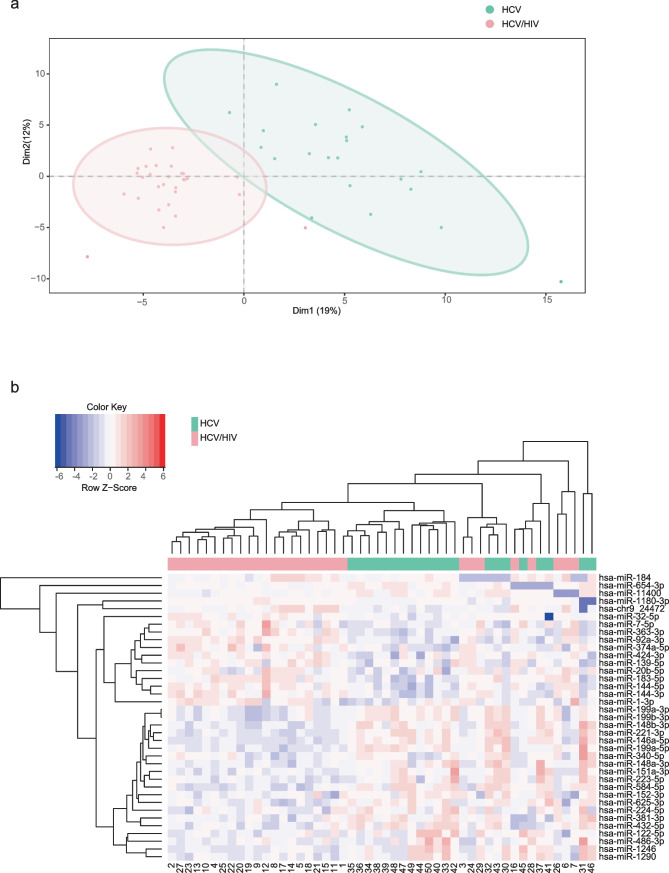
Analysis of EVs-microRNA cargo in HCV mono-infected vs HCV/HIV co-infected patients. **a** Exploratory analysis. Multivariate analysis was performed by supervised partial least squares discriminant analysis (PLS-DA) from normalized log transformed and scaled miRNA expression data. **b** Hierarchical cluster analysis of the SDE miRNAs. Study subjects are represented in columns and SDE miRNAs in rows, with clustering dendograms on the left for miRNAs and at the top for samples. The color scale shows the relative expression level of SDE miRNAs. Red color indicates a higher expression level and blue a lower expression level. Patients are grouped by infectious status

In the exploratory analysis, the PLS-DA of normalized miRNA counts showed a clear segregation of the HCV mono-infected samples from the HCV/HIV co-infected ones (Fig. 2a).

**Fig. 3:**
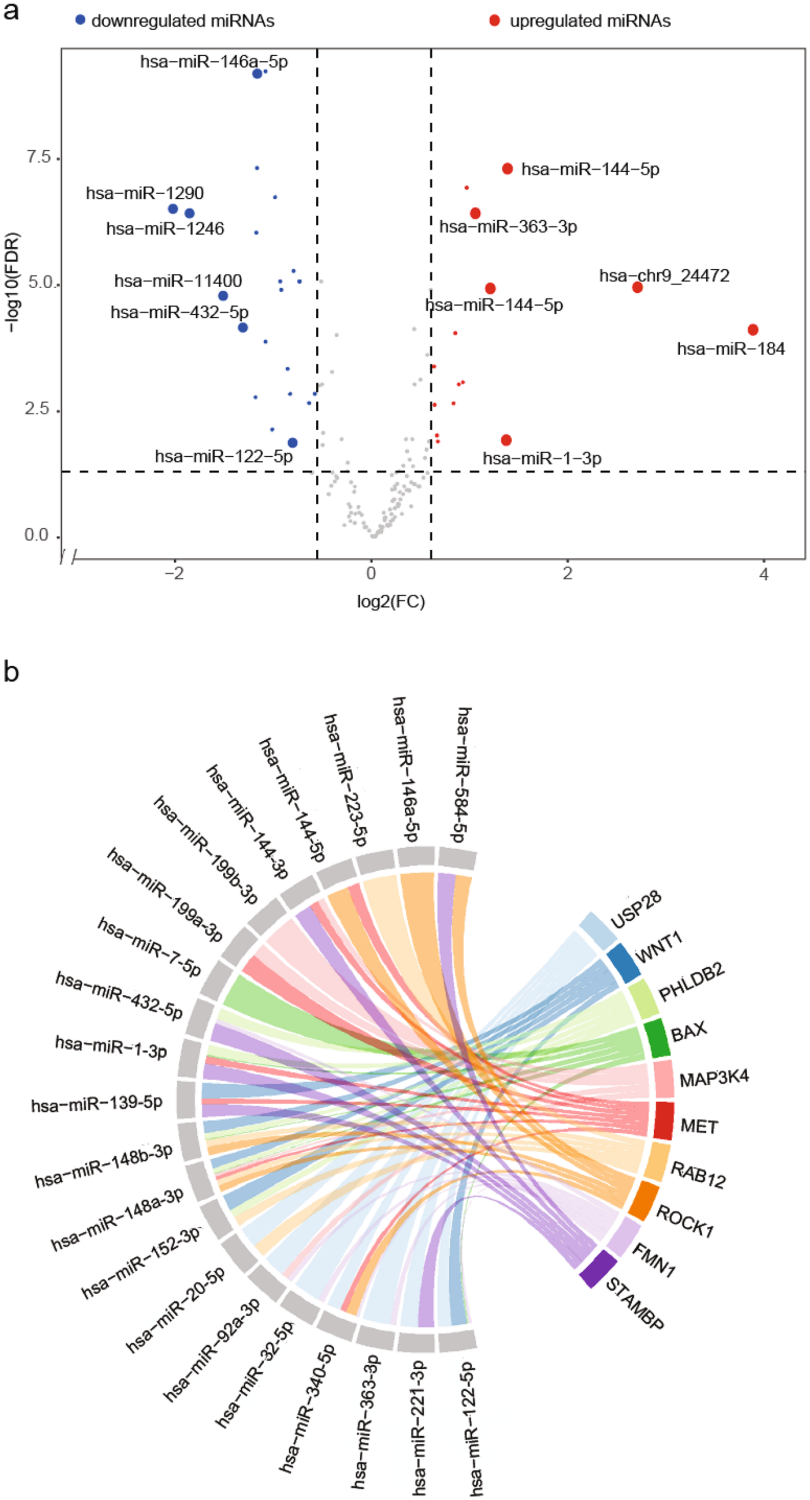
Differential miRNA expression analysis and target pathway analysis. **a** Volcano plot of SDE miRNAs. FDR false discovery rate, FC fold change. Red dots show miRNAs with a FDR corrected *p*-value ≤ 0.05 and a logFC ≥ 0.585 (equivalent to FC ≥ 1.5), blue dots show miRNAs with a FDR corrected *p*-value ≤ 0.05 and a |log FC|> 0.585, and gray dots show miRNAs without statistical significance. Highlighted dots are specially mentioned in the results section. **b** Chord diagram of the top 10 miRNA-gene interaction. Each targeted gene is represented by one different color, together with their corresponding interaction, while SDE miRNAs are shown on the right

The SDE analysis displayed 37 miRNAs (Fig. 2b and Supplementary Data 4). Fifteen miRNAs were upregulated in HCV/HIV co-infected group (Fig. 3a), including the putative de novo miRNA encoded by chromosome 9, which was strongly upregulated (hsa-chr9_24472; logFC = 2.69, FDR = 1.08E-06) (Supplementary Data 5). This putative miRNA is located at the q22.1 arm of chromosome 9 [chr9:87839044–87839112 (GRCh38/hg38)] within the intron 1 of the *fructose-bisphosphatase 2 pseudogene 1* (*FBP2P1*), coincident with an enhancer (ENSR00001151034) and a CTCF binding site, which has a key role in the regulation of miRNAs expression [19]. Among the upregulated miRNAs, the hsa-miR-184, hsa-miR-144-5p/3p, hsa-miR-1-3p, and hsa-miR-363-3p were highly expressed in the HCV/HIV co-infected cohort. On the other hand, 22 known miRNAs were downregulated in the HCV/HIV co-infected group, including hsa-miR-1290, hsa-miR-1246, hsa-miR-11400, hsa-miR-432-5p, and hsa-miR-146a-5p. Remarkably, the well-described hsa-miR-122-5p was also downregulated in this group.

Additionally, the area under the ROC curves (AUROC) was evaluated in order to assess whether SDE miRNAs correctly classify patients according to their infectious status. In this sense, eight of them showed an outstanding performance (AUROC > 0.9); moreover, two of them, namely the hsa-miR-146a-5p and hsa-miR-151a-3p, showed high abundance and an absolute logFC > 1.2 (Supplementary Data 4).

### Target enrichment analysis

The miRNA-gene interaction analysis showed 358 enriched target genes for the 37 SDE miRNAs. Supplementary Data 6 shows the top 50 target genes according to the FDR. We also explored SDE miRNA-gene interactions, and Fig. 3b shows the top ten. Note that three of the upregulated miRNAs significantly target over a hundred genes (hsa-miR-92a-3p, hsa-miR-32-5p, and hsa-miR-363-3p) (Supplementary Data 7).

In silico functional analysis showed that the significantly targeted genes are mostly involved in cancer-related pathways and cytokine mediated pathways (Supplementary Data 8). The top 25 pathways obtained by KEGG, REACTOME, and GO in the pathway-enrichment analysis for the SDE miRNAs is shown in Supplementary Data 9.

## Discussion

The analysis of the EVs presented here showed that while the concentration and size of EVs were similar, HCV coinfection with HIV produces a strong disturbance of miRNA content in EVs compared to the one observed in the HCV monoinfection. To our knowledge, this is the first study that characterizes the miRNA profile in plasma-EVs of HCV/HIV co-infected vs HCV mono-infected patients.

Scarce data is published about the size and concentration of plasma-derived EVs in HCV/HIV coinfection. In this study, the plasma-EVs’ characterization demonstrated that HIV coinfection does not significantly affect neither the EVs’ size nor concentration in a CHC setting. To date, EVs’ characterization remains a challenge; however, the techniques applied in this study are according to recent literature that suggests TEM as the most accurate approach to confirm the EVs’ structure and NTA to quantify their number and distribution [20]. In the studied cohorts, the isolated EVs’ size was compatible with small EVs according to MISEV guidelines [6]. The isolation procedure does not unequivocally purify specific types of vesicles but, rather, yield complex heterogeneous mixtures of EVs. It is also noteworthy to mention that the content of EV sub-fractions varies depending on the source of the EVs and/or the isolation techniques, which can ultimately largely influence the final results [21]. Therefore, although accumulating evidence in EVs’ composition are highly heterogeneous and dynamics, the correct control of methodological procedures and the use of appropriate protocols allowed us the further comparative analysis between groups [22, 23].

By performing high-throughput small RNA-Seq and computational profiling analyses, we described that the HCV/HIV co-infected group differentially expressed 37 miRNAs compared to the HCV mono-infected group. Some of these miRNAs were previously described to target genes associated with inflammation (cytokine-mediated pathways), fibrosis, and cancer development like USP28, WNT1, PHLDB2, BAX, MAP3K4, MET, RAB12, and ROCK1, among others. Additionally, some of them were reported to have a proviral effect in HCV life cycle, like hsa-miR-122, which was downregulated in the HCV/HIV co-infected group. Although it was formerly reported that HIV may promote HCV replication by enhancing the hsa-miR-122 in hepatoma cells, there is no previous data about its levels within EVs’ cargo between HCV/HIV co-infected vs HCV mono-infected patients [24]. We also detected in the HCV/HIV co-infected group a strong downregulation of miRNAs with antiviral effect, like hsa-miR-221, which accelerates anti-HCV treatment response, and hsa-miR-199a, that interacts directly with HCV genome to inhibit replication [25, 26]. Similarly, some others were described to participate in HIV life cycle, namely hsa-miR-1290 participates in HIV latency and hsa-miR-146a-5p is known to target CXCR4, an essential co-receptor in HIV entry route into T cells [27, 28]. Both miRNAs showed a deep downregulation in the HCV/HIV co-infected group of patients, which could be responsible of maintaining a higher viral transcription of HIV in CHC patients, as we have previously observed [29]. In this setting, the hsa-miR-146a-5p has been previously identified in plasma-EVs of HIV mono-infected patients, demonstrating a significantly higher expression than HIV-negative controls. This study from Chettimada et al. was performed with a similar methodological approach to our study, and interestingly, they did not observe differences between HCV/HIV co-infected and HIV mono-infected miRNA profiles [30].

It is important to mention that some of the identified differences could potentially be due to the impact of antiretroviral therapy. Although there are a limited number of studies in this field, a pilot study in rhesus macaques infected with the simian immunodeficiency virus (SIV) revealed that the antiretroviral therapy may modify both the abundance and the compartmentalization of several plasma EVs’ miRNA related to various diseases and biological processes [31]. However, it remains unknown if these differences are directly related to the therapy itself or are a consequence of the viral suppression. In this regard, it is worth mentioning that non-viremic and viremic HIV patients display slight differences in miRNA expression [32], these differences are mainly attributed to miRNAs with a reported role in HIV latency such as hsa-miR-29 family members, hsa-miRs -125b and -150. Additionally, differences in plasma miRNA profile have been identified between HIV mono-infected patients who respond or do not respond to ART [33]. Thus, considering that all HCV/HIV co-infected patients in our cohort were both on ART and achieved viral suppression, and bearing in mind previous evidence, we could suggest that the effect of ART does not significantly impact the miRNA profile.

Additionally, EVs carry different biological active molecules, which are key mediators in the progression of liver fibrosis and the subsequent development of HCC in the context of viral hepatitis (hepatitis A, B, C, and E) [8, 20]. Some of these 37 SDE miRNAs were associated with liver damage and HCV-induced HCC including hsa-miR-122-5p, hsa-miR-144, hsa-miR-1246, hsa-miR-224-5p, hsa-miR-221, hsa-miR-424-3p, hsa-miR-139-5p, hsa-miR-486-5p, and hsa-miR-199a family [34–41]. In line with this observation, the in silico pathway-enrichment analysis showed a strong presence of pathways related to cancer events, such as colorectal, breast, gastric, lung, melanoma, and pancreatic cancer as well as HCC, among others [8, 20]. Therefore, the signature of EVs arising from their cargo, especially miRNAs, plays an essential role in the outcome of the pathological processes.

Interestingly, differences in the liver miRNA expression profile between HCV/HIV co-infected and HCV mono-infected patients were recently described by Dalla et al. [42]. The existence of a distinct miRNA signature between these groups of patients in the liver microenvironment, as well as in the plasma-derived EVs, as we here described, strongly suggests the occurrence of different modulatory processes during coinfection. Moreover, the selective packaging of specific miRNAs into biologically active EVs, and its dissimilar profile from other biological materials, suggests a key role of plasma-derived EV miRNAs in early disease progression. In this line, Chunwen Pu et al. described an unlike expression of two HCC-related miRNAs (hsa-miR-21 and hsa-miR-144) in serum-EVs vs. EV-depleted serum, endorsing the idea of EVs as key mediators in the intercellular communication process [43]. Together, all this evidence indicates that the balance among the expression of distinct miRNAs would be the responsible for regulating disease progression.

The worst prognosis for the HCV/HIV coinfection could be partially explained by alterations in the dynamic network of interactions miRNA- target gene- protein, where EVs are key mediators. Therefore, the analysis of the EVs- miRNAs SDE between HCV/HIV co-infected and HCV mono-infected patients could facilitate the elucidation of these dissimilarities and, consequently, it will clarify our understanding of these conditions. Since miRNAs are the novel regulators of several crucial immunological and non-immunological processes, a clear comprehension of their role in antiviral immunity may allow the emergence of a new generation of biomarkers, in addition to the elucidation of the mechanisms driving virus-host interaction.

It should be held in mind that this study has certain limitations. First, we evaluated plasma-EVs, which is a mixture of EVs released by different cell types, not all of them infected by either HCV and/or HIV. Second, our study focused on small EVs. Large EVs including apoptotic bodies would provide information of a different metabolic stage of the cells meanwhile, small EVs have a greater potential as regulators of different molecular processes (although it is not exclusive to them). Third, the EVs characterization lack of the evaluation of EV-surface markers, an additional information that could be of interest to fully characterize this EVs, but the EV-miRNA composition is comparable with previous published reports. Fourth, the limited sample size could reduce our statistical power to detect smaller differences than 1.5 of fold change between the study groups. Fifth, additional possible confounders could be affecting our results. It may be important to acknowledge the potential influence of various factors such as environmental variables related to geographical differences on the observed outcomes. While we made efforts to control for these factors by balancing our populations for the most important clinical and biochemical variables related to HCV-disease, it is challenging to completely eliminate their influence. Sixth, this is an exploratory work, where only association was considered, and lacks mechanistic exploration or functional validation. Future studies, in a larger cohort, are essential to confirm virus–host interaction and disease progression. Moreover, it could be interesting to analyze EVs-miRNA from other populations such as healthy individuals, HIV mono-infected patients, and especially those cases with liver diseases such as CHC patients infected with hepatitis B virus.

## Conclusion

HCV/HIV coinfection does not affect the concentration or size of EVs but impacts the specific plasma derived-EVs miRNAs cargo. This signature for the HCV/HIV co-infected group demonstrated an in silico association with inflammation and cancer related pathways. These findings may help in the development of a new generation of biomarkers and treatment strategies, in addition to elucidate the mechanisms underlying virus-host interaction.

### Supplementary Information

Below is the link to the electronic supplementary material.

Supplementary file1 (DOCX 1028 KB)

## Data Availability

The datasets supporting the conclusions of this article are available in the ArrayExpress repository (EMBL-EBI) under accession number E-MTAB-11811 (EMBL-EBI; https://www.ebi.ac.uk/) and are included within the article and its supplementary file.
