# Supplementary material for: MicroRNA signature from extracellular vesicles of HCV/HIV co-infected individuals differs from HCV mono-infected
Source: J Mol Med (Berl). 2023 Sep 14;101(11):1409–20. doi: 10.1007/s00109-023-02367-8 (PMC10663177; doi:10.1007/s00109-023-02367-8)
Supplement: Supplementary file 1 — Supplementary file1 (DOCX 1028 KB) [file 109_2023_2367_MOESM1_ESM.docx]

**Supplementary data index**

**[Supplementary Data 1.](#_Supplementary_Data_1.)** [Extended](#_Supplementary_Data_1.)[data processing pipeline.](#_Supplementary_Data_1.)

[**Supplementary Data 2**. Microscopy characterization of plasma-derived extracellular vesicles from HCV mono-infected and HCV/HIV co-infected patients.](#_Supplementary_Data_3.)

# [**Supplementary Data 3.** Size range and concentration of plasma-derived extracellular vesicles from HCV mono-infected and HCV/HIV co-infected patients.](#_Supplementary_Data_4.)

[**Supplementary Data 4.** SDE miRNAs in HCV/HIV co-infected vs HCV mono-infected samples.](#_Supplementary_Data_5.)

[**Supplementary**](#_Supplementary_Data_5._1) **Data 5.** Novel miRNAs predicted by miRDeep.

# [**Supplementary Data 6.** MicroRNA-gene target enrichment analysis.](#_Supplementary_Data_6.)

[**Supplementary Data 7.** Barplot of the number of target genes by the SDE miRNAs in HCV/HIV co-infected patients](#_Supplementary_Data_7._1)

# [**Supplementary Data 8.** Heatmap pathway enrichment analysis.](#_Supplementary_Data_7.)

# [**Supplementary Data 9.** Top 25 pathways obtained in the pathway-enrichment analysis target genes of SDE miRNAs.](#_Supplementary_Data_7B9.)

**Supplementary data**

# **Supplementary Data 1.** Extended data processing pipeline.

Bioinformatics Analysis.

Raw data were analyzed with a specific bioinformatic pipeline for the identification of known and novel miRNAs. Quality control was performed with FastQC (v.0.11.3); adapter sequences as well as low-quality base calls (q < 20) were trimmed with cutadapt (v.1.13) and processed with miRDeep2 (v.0.0.7). This software allows the alignment of the reads to the reference human genome (GRCh38) using the mapper.pl module based on Bowtie1. Only the alignments with zero mismatches in the seed region and those that did not map to more than five different loci in the genome were retained. Quantification of miRNAs was performed with the quantifier.pl module, which determines the expression of the corresponding known miRNAs in two steps. First, predefined mature miRNA sequences are mapped against the reference precursors in miRBase (v21), a public repository miRNA which contains 2888 human miRNAs sequences [1]. Second, sequencing reads were mapped against the precursor sequences. Reads falling into an interval of two nucleotides upstream and five nucleotides downstream of the mature miRNA sequences were determined for quantification.

Statistical analysis

For data preprocessing and exploratory analysis, first, miRNAs with low read counts were filtered out by the filterByExpr edgeR function, retaining only those miRNAs that have sufficiently large counts according to the minimum expression criterion [2]. miRNA count matrix was normalized with Trimmed Mean of M-values (TMM) and count per million (CPM) approaches for library size normalization. Then, for exploration analysis, partial least square discriminant analysis (PLS-DA) with the normalized miRNAs counts was performed.

Fort the significant differentially expressed (SDE) miRNA analysis, miRNA expression differences between groups were analyzed using a binomial negative generalized linear model (bnGLM). MiRNAs with a fold change (FC) ≥ 1.5 and an adjusted *p*-value ≤ 0.05 using the false discovery rate (FDR) were considered SDE miRNAs.

miRNA-based target prediction and pathway enrichment analysis

Finally, SDE miRNA-target interactions and pathway enrichment analysis of the target genes was performed. Only experimentally validated interactions were considered. Enriched target genes were considered with at least four validated interactions with SDE miRNAs and enrichment *p*-values corrected for false discovery rate (FDR) ≤ 0.05. Functional enrichment analysis of the enriched target genes were analyzed by means of three databases: GO, KEGG and REACTOME. Enrichment *p*-values (Fischer’s exact test with hypergeometric distribution) were corrected for the false discovery rate (FDR) (*q value* ≤ 0.05).

# **Supplementary Data 2.** Microscopy characterization of plasma-derived extracellular vesicles from HCV mono-infected and HCV/HIV co-infected patients.

Representative images of the isolated plasma-derived vesicles by Transmission electron microscopy (TEM) (**a**) and cryo-electron microscopy (Cryo-EM) (**b**)

**
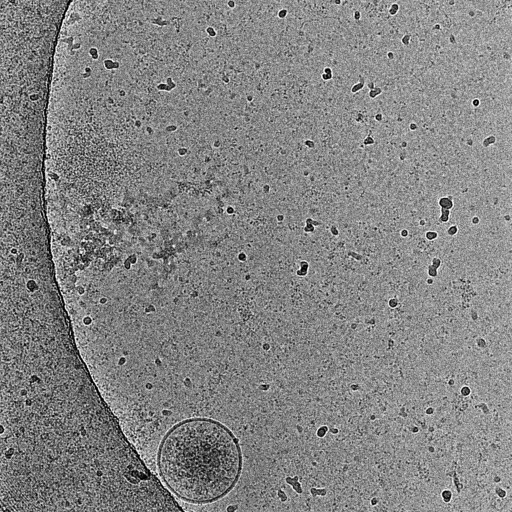

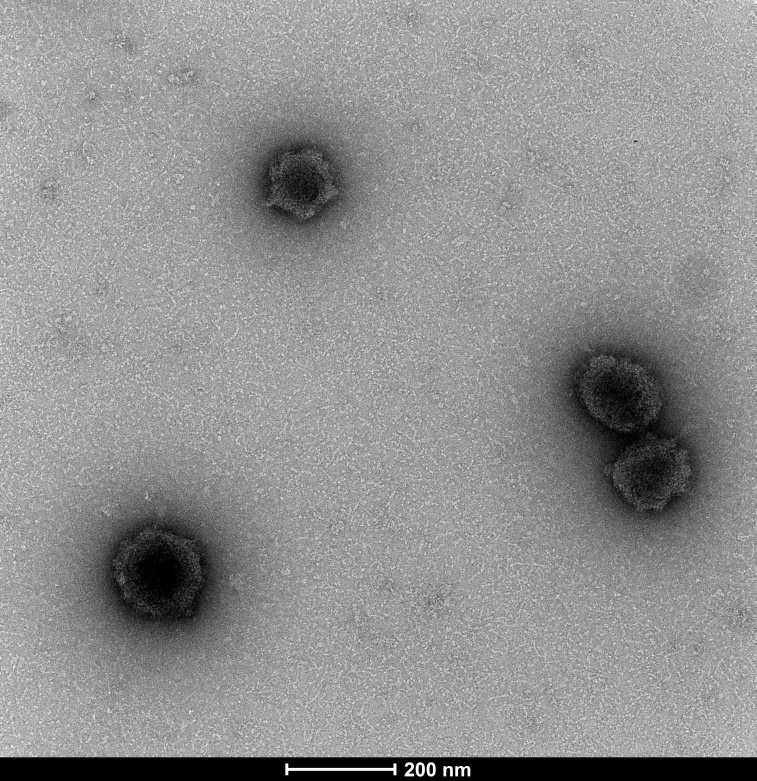
**

b

100 nm

200 nm

# **Supplementary Data 3.** Size range and concentration of plasma-derived extracellular vesicles from HCV mono-infected and HCV/HIV co-infected patients.

Boxplot of particle sizes (**a**) and concentration (**b**) measured with NTA according to infection condition. HCV mono-infected group represented in light-blue and HCV/HIV co-infected in light-pink. Horizontal lines within boxes indicate medians. Horizontal lines outside the boxes represent the 5 and 95 percentiles. Mean is indicated as a dark-red diamond. The Mann-Whitney *U* test was used to compare sets of data. The table details size and concentration median (percentile 25; percentile 75) and *p*-values for the comparison between HCV mono-infected and HCV/HIV co-infected results.


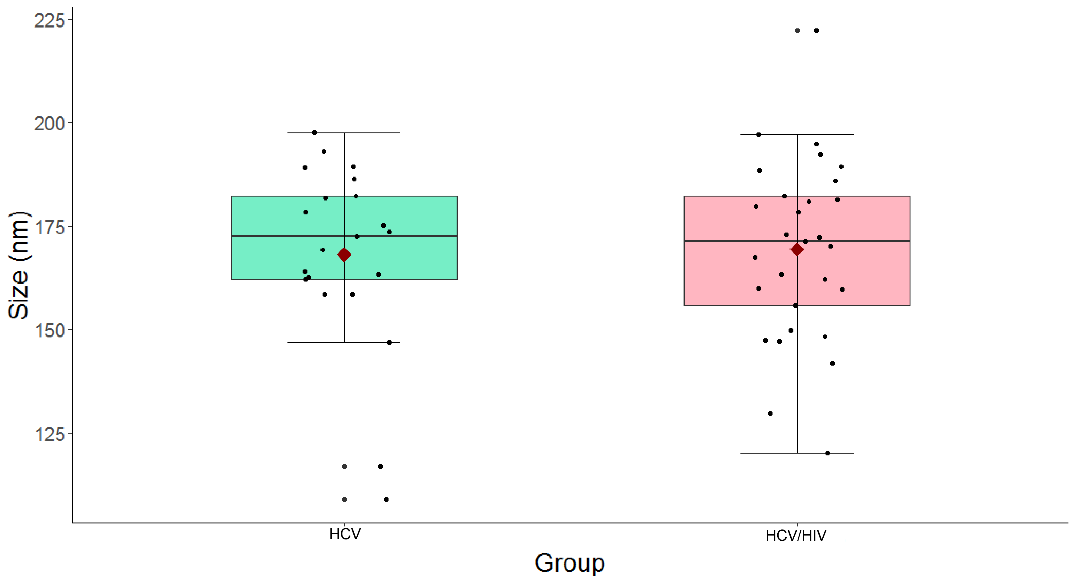


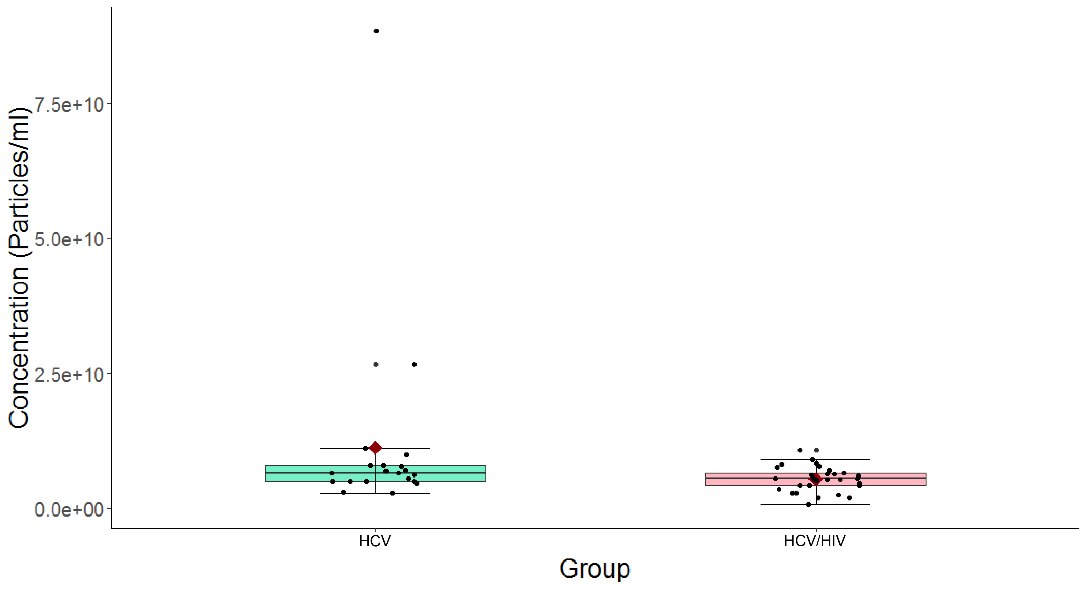


| **NTA** | **Size (nm)** | ***P* value** | **Concentration (particles/ml)** | ***P* value** |
| --- | --- | --- | --- | --- |
| **HCV samples** | 172.4 (160.3; 184.3) | 0.888 | 6.5E09 (5E09;8E09) | 0.064 |
| **HCV/HIV samples** | 171.3 (152.8; 184.1) |  | 5.5E09 (4.1E9;7.1E09) |  |

**Supplementary Data 4.** SDE miRNAs in HCV/HIV co-infected vs HCV mono-infected samples.

| **Downregulated miRNAs** | | | | | |
| --- | --- | --- | --- | --- | --- |
| **miRNA** | **logFC** | **FDR** | **HCV counts** | **HCV/HIV counts** | **AUROC** |
| **hsa-miR-1290** | -2.04944 | 3.06E-07 | 342.7 (179.2-918.2) | 113.6 (63.7-175.8) | 0.8571 |
| **hsa-miR-1246** | -1.87948 | 3.51E-07 | 1026.0 (550.3-2319.0) | 325.0 (258.6-548.1) | 0.8144 |
| **hsa-miR-11400** | -1.54928 | 1.46E-05 | 183.3 (74.4-214.1) | 39.1 (20.5-84.7) | 0.7455 |
| **hsa-miR-432-5p** | -1.33714 | 6.58E-05 | 195.8 (60.8-316.5) | 83.5 (40.1-101.2) | 0,7635 |
| **hsa-miR-146a-5p** | -1.22368 | 6.51E-10 | 13125.0 (10739.0-20933.0) | 5890.0 (5428.0-6991.0) | 0.9622 |
| **hsa-miR-486-3p** | -1.2106 | 0.001654 | 290.6 (172.6-534.5) | 240.3 (182.9-296.5) | 0.6256 |
| **hsa-miR-148a-3p** | -1.19943 | 9.11E-07 | 22631.0 (14810.0-38118.0) | 11283.0 (9646.0-12923.0) | 0.8621 |
| **has-miR-199a-5p** | -1.19191 | 4.78E-08 | 1320.0 (797.9-1896.0) | 639.9 (491.8-734.4) | 0.8456 |
| **hsa-miR-381-3p** | -1.10608 | 0.000132 | 438.2 (218.8-772.7) | 233.7 (156.1-286.3) | 0.7438 |
| **hsa-miR-221-3p** | -1.10598 | 5.80E-10 | 2182.0 (1752.0-3312.0) | 1036.0 (912.3-1325.0) | 0.9573 |
| **hsa-miR-654-3p** | -1.03753 | 0.007296 | 183.3 (76.9-329.6) | 96.7 (41.3-154.3) | 0.6683 |
| **hsa-miR-148b-3p** | -1.0098 | 1.80E-07 | 1314.0 (691.7-1580.0) | 585.7 (481.3-657.2) | 0.8637 |
| **hsa-miR-584-5p** | -0.95697 | 8.41E-06 | 412.7 (262.4-511.3) | 176.0 (137.5-241.9) | 0.8949 |
| **hsa-miR-223-5p** | -0.94603 | 1.24E-05 | 1186.0 (756.9-1506.0) | 601.6 (439.6-683.4) | 0.8473 |
| **hsa-miR-625-3p** | -0.88129 | 0.000455 | 304.5 (192.6-440.8) | 157.1 (103.5-225.5) | 0.8161 |
| **hsa-miR-224-5p** | -0.85655 | 0.001422 | 312.1 (228.1-638.0) | 217.5 (145.5-340.2) | 0.7143 |
| **hsa-miR-122-5p** | -0.83147 | 0.013329 | 53183.0 (31102.0-113228.0) | 22100.0 (17917.0-41783.0) | 0.7455 |
| **hsa-miR-151a-3p** | -0.8204 | 5.23E-06 | 13079.0 (10279.0-18786.0) | 8502.0 (7715.0-9591.0) | 0.9146 |
| **hsa-miR-199a-3p** | -0.75996 | 8.41E-06 | 1259.0 (1056.0-1492.0) | 676.8 (545.5-909.0) | 0.9113 |
| **hsa-miR-199b-3p** | -0.75979 | 8.41E-06 | 1259.0 (1056.0-1492.0) | 683.3 (536.5-909.0) | 0.9080 |
| **hsa-miR-340-5p** | -0.6626 | 0.002167 | 575.1 (358.5-869.3) | 388.0 (320.8-543.3) | 0.6831 |
| **hsa-miR-152-3p** | -0.60514 | 0.00142 | 334.4 (272.3-412.7) | 225.6 (143.8-287.1) | 0.8161 |

| **Upregulated miRNAs** | | | | | | | | | |
| --- | --- | --- | --- | --- | --- | --- | --- | --- | --- |
| **miRNA** | **logFC** | | **FDR** | | **HCV counts** | | **HCV/HIV counts** | | **AUROC** |
| **hsa-miR-184** | | 3.856673 | | 6.79E-05 | | 35.20 (15.6-73.4) | | 47 (20.9-290.9) | 0.5928 |
| **hsa-chr9_24472** | | 2.693477 | | 1.01E-05 | | 135.3 (75.7-194.4) | | 291.4 (167.8-1739.0) | 0.8325 |
| **hsa-miR-144-5p** | | 1.382283 | | 4.78E-08 | | 548.3 (372.7-1013.0) | | 1582.0 (1329.0-1924.0) | 0.9458 |
| **hsa-miR-1-3p** | | 1.353063 | | 0.010282 | | 104.5 (74.4-223.4) | | 379.6 (94.2-561.7) | 0.7028 |
| **hsa-miR-144-3p** | | 1.180373 | | 1.10E-05 | | 1023.0 (650.9-1704.0) | | 2308.0 (1757.0-3223.0) | 0.8571 |
| **hsa-miR-363-3p** | | 1.031806 | | 3.51E-07 | | 3332.0 (2764.0-3884.0) | | 6166.0 (4581.0-7550.0) | 0.9146 |
| **hsa-miR-92a-3p** | | 0.942827 | | 1.17E-07 | | 6334.0 (4740.0-7342.0) | | 11965.0 (9459.0-13858.0) | 0.9179 |
| **hsa-miR-183-5p** | | 0.908059 | | 0.000838 | | 584.5 (304.8-949.8) | | 1301.0 (806.0-1502.0) | 0.8259 |
| **hsa-miR-32-5p** | | 0.86405 | | 0.000931 | | 143.6 (105.9-223.0) | | 229.7 (178.6-356.8) | 0.7668 |
| **hsa-miR-424-3p** | | 0.826871 | | 8.95E-05 | | 134.7 (75.7-163.5) | | 204.2 (157.4-234.8) | 0.8325 |
| **hsa-miR-1180-3p** | | 0.809772 | | 0.002199 | | 91.4 (63.7-151.2) | | 162.2 (122.3-203.1) | 0.7553 |
| **hsa-miR-139-5p** | | 0.648897 | | 0.012625 | | 2555.0 (1506.0-3683.0) | | 4224.0 (3351.0-5385.0) | 0.7816 |
| **hsa-miR-20b-5p** | | 0.641284 | | 0.009494 | | 119.2 (97.8-209.2) | | 216.2 (151.3-281.4) | 0.6897 |
| **hsa-miR-374a-5p** | | 0.616556 | | 0.002355 | | 344.5 (235.7-446.2) | | 500.5 (361.4-653.3) | 0.7241 |
| **hsa-miR-7-5p** | | 0.610916 | | 0.000407 | | 2399.0 (2086.0-2932.0) | | 3365.0 (2608.0-3981.0) | 0.7668 |

Each column represent: the SDE miRNA identifier (miRBase format), the LogFC indicating the expression fold change between HCV/HIV co-infected and HCV mono-infected group, the adjusted *p*-value (FDR, corrected for multiple testing with the Benjamini– Hochberg procedure), abundance of miRNA for HCV and HCV/HIV samples expressed as median count (percentile 25; percentile 75) and area under the ROC curves (AUROC).

# **Supplementary Data 5.** Novel miRNAs predicted by miRDeep.

Details of the putative *de novo* miRNAs.

| **Provisional id** | **miRDeep2 score** | **Estimated probability that the miRNA candidate is a true positive** | **Mature read count** | **Star read count** | **Significant randfold p-value** | **Consensus mature sequence** |
| --- | --- | --- | --- | --- | --- | --- |
| chr9_24472 | 4.9 | 28 +/- 11% | 591 | 0 | yes | ugggugguggugcaugucu |

Column 1 represents the identifier generated by mirdeep2 according to the genomic location of the identified de novo RNAs; column 2-6 represent parameters estimated by miRDeep2 according to the probability of being a true miRNA, its counts, the RANDFOLD test (calculates the free energy of folding [secondary structure] of the miRNA to determine the possibility of being a miRNA) and its sequence.

Secondary structure of the microRNA *de novo* chr9_24472 determined with mirdeep2.


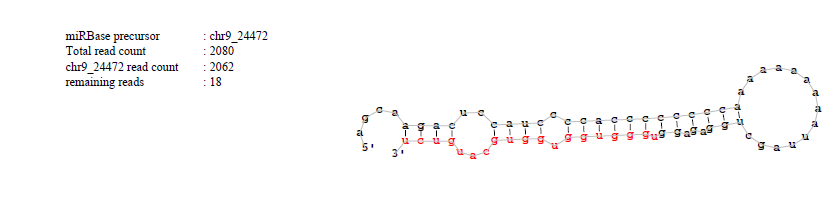

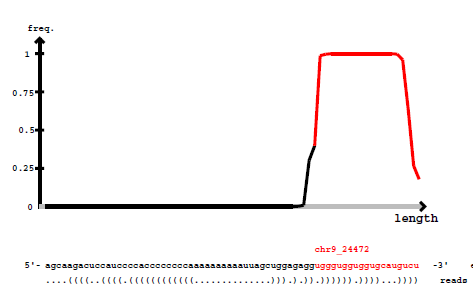


This putative miRNA is located at the q22.1 arm of chromosome 9 [chr9:87839044-87839112 (GRCh38/hg38)] within the intron 1 of the fructose-bisphosphatase 2 pseudogene 1 (FBP2P1), coincident with an enhancer (ENSR00001151034) and a CTCF binding site.


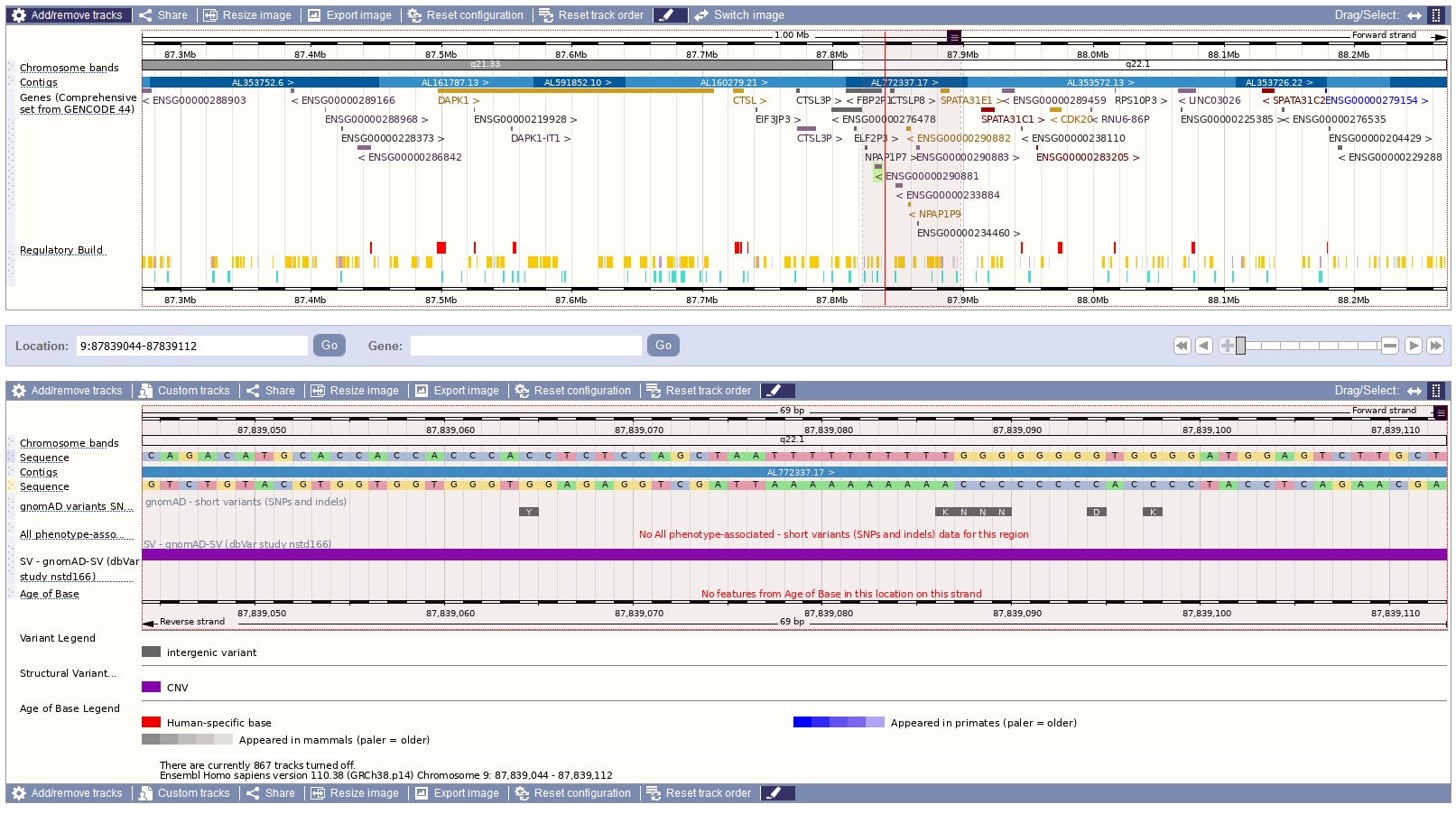


Zoom in into this region:


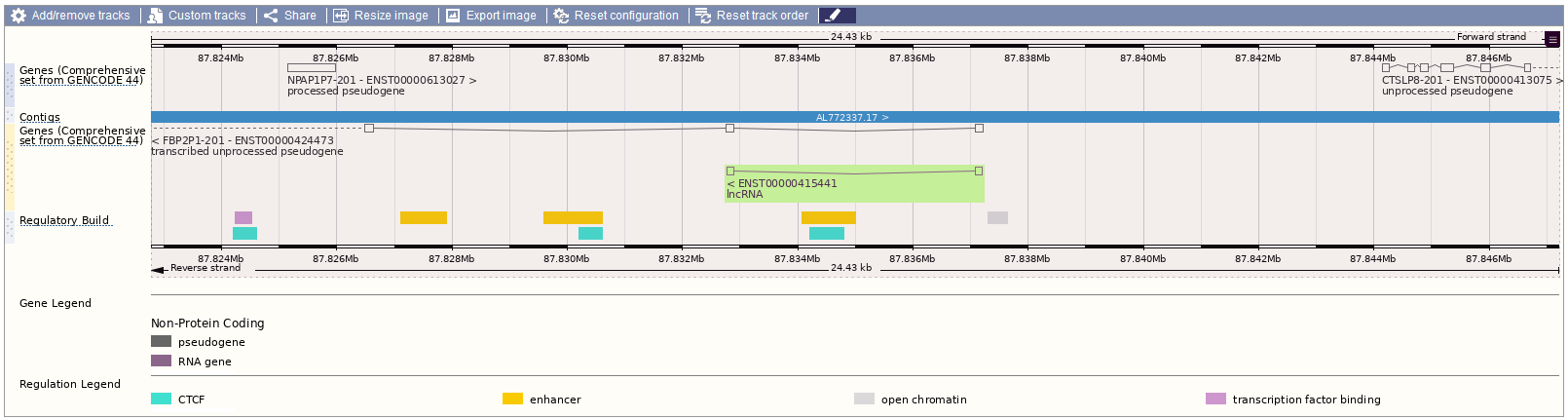


Images from <http://www.ensembl.org/Homo_sapiens>

# **Supplementary Data 6.** MicroRNA-gene target enrichment analysis.

Top 50 genes according to the FDR obtained in the target-enrichment analysis for the SDE miRNAs.

| **Gene symbol** | **Function involved** | **Number of interactions** | **miRNAs** | **FDR** |
| --- | --- | --- | --- | --- |
| **USP28** | DNA damage response/ Cancer | 7 | hsa-miR-122-5p / hsa-miR-221-3p / hsa-miR-363-3p / hsa-miR-340-5p / hsa-miR-32-5p / hsa-miR-92a-3p / hsa-miR-20b-5p | 3.2344E-05 |
| **WNT1** | Developmental processes/ Inflammation/ Cancer | 5 | hsa-miR-122-5p / hsa-miR-152-3p / hsa-miR-148a-3p / hsa-miR-148b-3p / hsa-miR-139-5p | 3.2344E-05 |
| **PHLDB2** | Cytoskeleton organization/ Embryonic development regulation/ Cancer | 5 | hsa-miR-1-3p / hsa-miR-432-5p / hsa-miR-148a-3p / hsa-miR-148b-3p / hsa-miR-152-3p | 3.6609E-05 |
| **BAX** | Apoptosis | 5 | hsa-miR-148b-3p / hsa-miR-122-5p / hsa-miR-1-3p / hsa-miR-7-5p / hsa-miR-148a-3p | 5.4864E-05 |
| **MAP3K4** | Cancer | 5 | hsa-miR-148a-3p / hsa-miR-92a-3p / hsa-miR-199a-3p / hsa-miR-199b-3p / hsa-miR-144-3p | 5.4864E-05 |
| **MET** | Cellular survival/ Embryogenesis/ Cellular migration and invasion/ Cancer | 7 | hsa-miR-1-3p / hsa-miR-199a-3p / hsa-miR-340-5p / hsa-miR-148a-3p / hsa-miR-144-3p / hsa-miR-139-5p / hsa-miR-144-5p | 5.4864E-05 |
| **RAB12** | Cellular response to insulin stimulus/ Autophagy/ Cancer | 5 | hsa-miR-148a-3p / hsa-miR-20b-5p / hsa-miR-152-3p / hsa-miR-148b-3p / hsa-miR-223-5p | 5.4864E-05 |
| **ROCK1** | Cytoskeleton organization/  Angiogenesis regulation/ cellular motility and invasion/ Cancer | 6 | hsa-miR-146a-5p / hsa-miR-584-5p / hsa-miR-148b-3p / hsa-miR-148a-3p / hsa-miR-340-5p / hsa-miR-144-5p | 5.4864E-05 |
| **FMN1** | Cytoskeleton organization | 6 | hsa-miR-432-5p / hsa-miR-340-5p / hsa-miR-92a-3p / hsa-miR-363-3p / hsa-miR-32-5p / hsa-miR-122-5p | 0.00012102 |
| **STAMBP** | Endosome-lysosome pathway/ Stability of cell substrates/ Cell growth/ Cytokine-mediated signaling/ Cell cycle progression/ Cancer | 6 | hsa-miR-221-3p / hsa-miR-139-5p / hsa-miR-584-5p / hsa-miR-1-3p / hsa-miR-432-5p / hsa-miR-144-3p | 0.00012102 |
| **BAZ2B** | Human hematopoietic cells reprogramming control | 6 | hsa-miR-92a-3p / hsa-miR-32-5p / hsa-miR-363-3p / hsa-miR-152-3p / hsa-miR-148b-3p / hsa-miR-148a-3p | 0.00026214 |
| **CNEP1R1** | Triacylglycerol synthesis | 6 | hsa-miR-32-5p / hsa-miR-92a-3p / hsa-miR-363-3p / hsa-miR-199a-3p / hsa-miR-199b-3p / hsa-miR-144-3p | 0.00026214 |
| **CNOT4** | Transcriptional regulation | 7 | hsa-miR-152-3p / hsa-miR-148b-3p / hsa-miR-148a-3p / hsa-miR-92a-3p / hsa-miR-363-3p / hsa-miR-32-5p / hsa-miR-20b-5p | 0.00026214 |
| **GFPT2** | [Insulin resistance](https://www.genome.jp/kegg-bin/show_pathway?hsa04931/GFPT2/default%3dpink) | 5 | hsa-miR-32-5p / hsa-miR-92a-3p / hsa-miR-363-3p / hsa-miR-199b-3p / hsa-miR-199a-3p | 0.00026214 |
| **MECP2** | Mammalian embryonic development/ Neurologic disorder | 8 | hsa-miR-199a-3p / hsa-miR-122-5p / hsa-miR-199a-5p / hsa-miR-148b-3p / hsa-miR-92a-3p / hsa-miR-340-5p / hsa-miR-432-5p / hsa-miR-20b-5p | 0.00026214 |
| **ZIC5** | Transcriptional repression/ Cancer | 8 | hsa-miR-340-5p / hsa-miR-7-5p / hsa-miR-152-3p / hsa-miR-148b-3p / hsa-miR-148a-3p / hsa-miR-92a-3p / hsa-miR-363-3p / hsa-miR-32-5p | 0.00026214 |
| **MYLIP** | Cholesterol homeostasis | 9 | hsa-miR-20b-5p / hsa-miR-92a-3p / hsa-miR-340-5p / hsa-miR-32-5p / hsa-miR-363-3p / hsa-miR-221-3p / hsa-miR-224-5p / hsa-miR-374a-5p / hsa-miR-654-3p | 0.00031364 |
| **SMAD4** | Cell growth regulation/ Angiogenesis/ Apoptosis/ Cell invasion/ Cancer | 8 | hsa-miR-92a-3p / hsa-miR-146a-5p / hsa-miR-224-5p / hsa-miR-199a-5p / hsa-miR-183-5p / hsa-miR-20b-5p / hsa-miR-144-3p / hsa-miR-144-5p | 0.00038023 |
| **LHFPL2** | Predicted to be involved in development of primary sexual characteristics | 7 | hsa-miR-221-3p / hsa-miR-32-5p / hsa-miR-374a-5p / hsa-miR-223-5p / hsa-miR-92a-3p / hsa-miR-363-3p / hsa-miR-224-5p | 0.00038404 |
| **TRAM2** | Reticulum endoplasmic posttranslational secretory control | 6 | hsa-miR-122-5p / hsa-miR-7-5p / hsa-miR-424-3p / hsa-miR-92a-3p / hsa-miR-363-3p / hsa-miR-32-5p | 0.00038404 |
| **PLPP4** | Cancer | 4 | hsa-miR-199a-5p / hsa-miR-152-3p / hsa-miR-148b-3p / hsa-miR-148a-3p | 0.00038794 |
| **ACTC1** | Cell motility/ Cardiomyopathy | 7 | hsa-miR-1-3p / hsa-miR-139-5p / hsa-miR-32-5p / hsa-miR-340-5p / hsa-miR-363-3p / hsa-miR-7-5p / hsa-miR-92a-3p | 0.00042088 |
| **ITGA5** | Tumor invasion/ Cancer | 5 | hsa-miR-92a-3p / hsa-miR-148b-3p / hsa-miR-183-5p / hsa-miR-152-3p / hsa-miR-148a-3p | 0.00042088 |
| **REV3L** | DNA synthesis/damage | 5 | hsa-miR-340-5p / hsa-miR-183-5p / hsa-miR-32-5p / hsa-miR-92a-3p / hsa-miR-363-3p | 0.00046578 |
| **SESN3** | Blood glucose regulation/ Insulin resistance/ lipid storage in obesity | 9 | hsa-miR-374a-5p / hsa-miR-363-3p / hsa-miR-92a-3p / hsa-miR-32-5p / hsa-miR-20b-5p / hsa-miR-584-5p / hsa-miR-148a-3p / hsa-miR-148b-3p / hsa-miR-152-3p | 0.00046578 |
| **UHRF1** | Gene expression regulation/ p53-dependent DNA damage checkpoint/ Cancer | 4 | hsa-miR-1-3p / hsa-miR-146a-5p / hsa-miR-7-5p / hsa-miR-221-3p | 0.00046578 |
| **CCT6A** | Cancer | 5 | hsa-miR-32-5p / hsa-miR-148a-3p / hsa-miR-152-3p / hsa-miR-148b-3p / hsa-miR-625-3p | 0.00051727 |
| **ATP7A** | Copper transportation | 5 | hsa-miR-148b-3p / hsa-miR-122-5p / hsa-miR-32-5p / hsa-miR-92a-3p / hsa-miR-223-5p | 0.00059278 |
| **SLC7A11** | Mediator of Kaposi sarcoma-associated herpesvirus fusion and cell entry | 8 | hsa-miR-122-5p / hsa-miR-148b-3p / hsa-miR-340-5p / hsa-miR-32-5p / hsa-miR-92a-3p / hsa-miR-363-3p / hsa-miR-223-5p / hsa-miR-20b-5p | 0.00060268 |
| **ANKIB1** | Proteasomal ubiquitin-dependent protein catabolic process Regulation | 4 | hsa-miR-1-3p / hsa-miR-20b-5p / hsa-miR-32-5p / hsa-miR-92a-3p | 0.00062965 |
| **PIK3CD** | Immune response | 5 | hsa-miR-7-5p / hsa-miR-199a-5p / hsa-miR-32-5p / hsa-miR-363-3p / hsa-miR-92a-3p | 0.00062965 |
| **PPARD** | Transcriptional repression and nuclear receptor signaling/ Cancer | 4 | hsa-miR-92a-3p / hsa-miR-148a-3p / hsa-miR-148b-3p / hsa-miR-152-3p | 0.00062965 |
| **ATXN1** | Immunomodulation/ Neurodegenerative disorders | 9 | hsa-miR-340-5p / hsa-miR-221-3p / hsa-miR-32-5p / hsa-miR-92a-3p / hsa-miR-374a-5p / hsa-miR-1290 / hsa-miR-144-3p / hsa-miR-20b-5p / hsa-miR-7-5p | 0.0006793 |
| **BCL11A** | Fetal hemoglobin silencing in adult cells/ B-cell malignancies | 4 | hsa-miR-148b-3p / hsa-miR-340-5p / hsa-miR-32-5p / hsa-miR-486-3p | 0.00070532 |
| **NFKB1** | Wide variety of biological functions/ Immune cell development/ the immediate-early response to viral infection | 4 | hsa-miR-146a-5p / hsa-miR-92a-3p / hsa-miR-199a-5p / hsa-miR-139-5p | 0.00070532 |
| **SLC39A6** | Protein, nucleic acid, carbohydrate, and lipid metabolism/ Gene transcription control/ Growth, development/ Cell differentiation | 4 | hsa-miR-32-5p / hsa-miR-139-5p / hsa-miR-144-3p / hsa-miR-92a-3p | 0.00070532 |
| **ZCCHC2** | Predicted to have nucleic acid binding activity and zinc ion binding activity | 6 | hsa-miR-148b-3p / hsa-miR-340-5p / hsa-miR-122-5p / hsa-miR-92a-3p / hsa-miR-144-3p / hsa-miR-374a-5p | 0.00070532 |
| **MCOLN2** | Predicted to have cation channel activity | 5 | hsa-miR-32-5p / hsa-miR-92a-3p / hsa-miR-199b-3p / hsa-miR-199a-3p / hsa-miR-363-3p | 0.00070659 |
| **FXR1** | Organ development; muscle fiber development; and somitogenesis | 7 | hsa-miR-32-5p / hsa-miR-92a-3p / hsa-miR-7-5p / hsa-miR-148a-3p / hsa-miR-148b-3p / hsa-miR-151a-3p / hsa-miR-152-3p | 0.00079126 |
| **HIVEP1** | Transcriptional regulation of both HIV and cellular genes | 4 | hsa-miR-221-3p / hsa-miR-32-5p / hsa-miR-363-3p / hsa-miR-92a-3p | 0.00079126 |
| **MORC3** | Epigenetic gene silencing/ Developmental abnormalities/ Cancers | 4 | hsa-miR-32-5p / hsa-miR-584-5p / hsa-miR-92a-3p / hsa-miR-144-3p | 0.00079126 |
| **SNAPIN** | insulin secretion regulation/ Lysosomes and autophagy maintenance in macrophages | 5 | hsa-miR-1-3p / hsa-miR-7-5p / hsa-miR-148a-3p / hsa-miR-152-3p / hsa-miR-148b-3p | 0.00079126 |
| **GAS1** | Tumor suppression | 5 | hsa-miR-183-5p / hsa-miR-148a-3p / hsa-miR-152-3p / hsa-miR-148b-3p / hsa-miR-184 | 0.00083771 |
| **DNAJC27** | Predicted to be involved in intracellular protein transport and positive regulation of MAPK cascade | 4 | hsa-miR-7-5p / hsa-miR-32-5p / hsa-miR-92a-3p / hsa-miR-20b-5p | 0.00090638 |
| **MAPK9** | Proliferation, differentiation, transcription regulation and development/ Cell death | 4 | hsa-miR-199a-3p / hsa-miR-7-5p / hsa-miR-92a-3p / hsa-miR-20b-5p | 0.00090638 |
| **SOS2** | Positive regulation of ras proteins | 4 | hsa-miR-1-3p / hsa-miR-152-3p / hsa-miR-148b-3p / hsa-miR-148a-3p | 0.00090638 |
| **PMEPA1** | Androgens and transforming growth factor beta induction/ Cancer | 6 | hsa-miR-7-5p / hsa-miR-654-3p / hsa-miR-374a-5p / hsa-miR-92a-3p / hsa-miR-32-5p / hsa-miR-363-3p | 0.00096528 |
| **PTAR1** | Predicted to have protein prenyltransferase activity | 6 | hsa-miR-1-3p / hsa-miR-7-5p / hsa-miR-374a-5p / hsa-miR-32-5p / hsa-miR-363-3p / hsa-miR-92a-3p | 0.00096528 |
| **B4GALT7** | Glycosaminoglycan synthesis | 6 | hsa-miR-152-3p / hsa-miR-148b-3p / hsa-miR-148a-3p / hsa-miR-32-5p / hsa-miR-363-3p / hsa-miR-92a-3p | 0.00097395 |
| **HECTD1** | Organ development/ Negative regulation of protein localization to plasma membrane; and protein autoubiquitination | 4 | hsa-miR-221-3p / hsa-miR-374a-5p / hsa-miR-32-5p / hsa-miR-92a-3p | 0.00097395 |

Each column represent: the target gene symbol (Gene symbol), function involved, the number of SDE miRNAs that modulate each enriched gene (Number of interactions), the miRNA identifier in miRBase format and the adjusted *p*-value (FDR, corrected *p*-value for multiple testing performed with the Benjamini–Hochberg procedure).

# **Supplementary Data 7.** Barplot of the number of target genes by the SDE miRNAs in HCV/HIV co-infected patients.


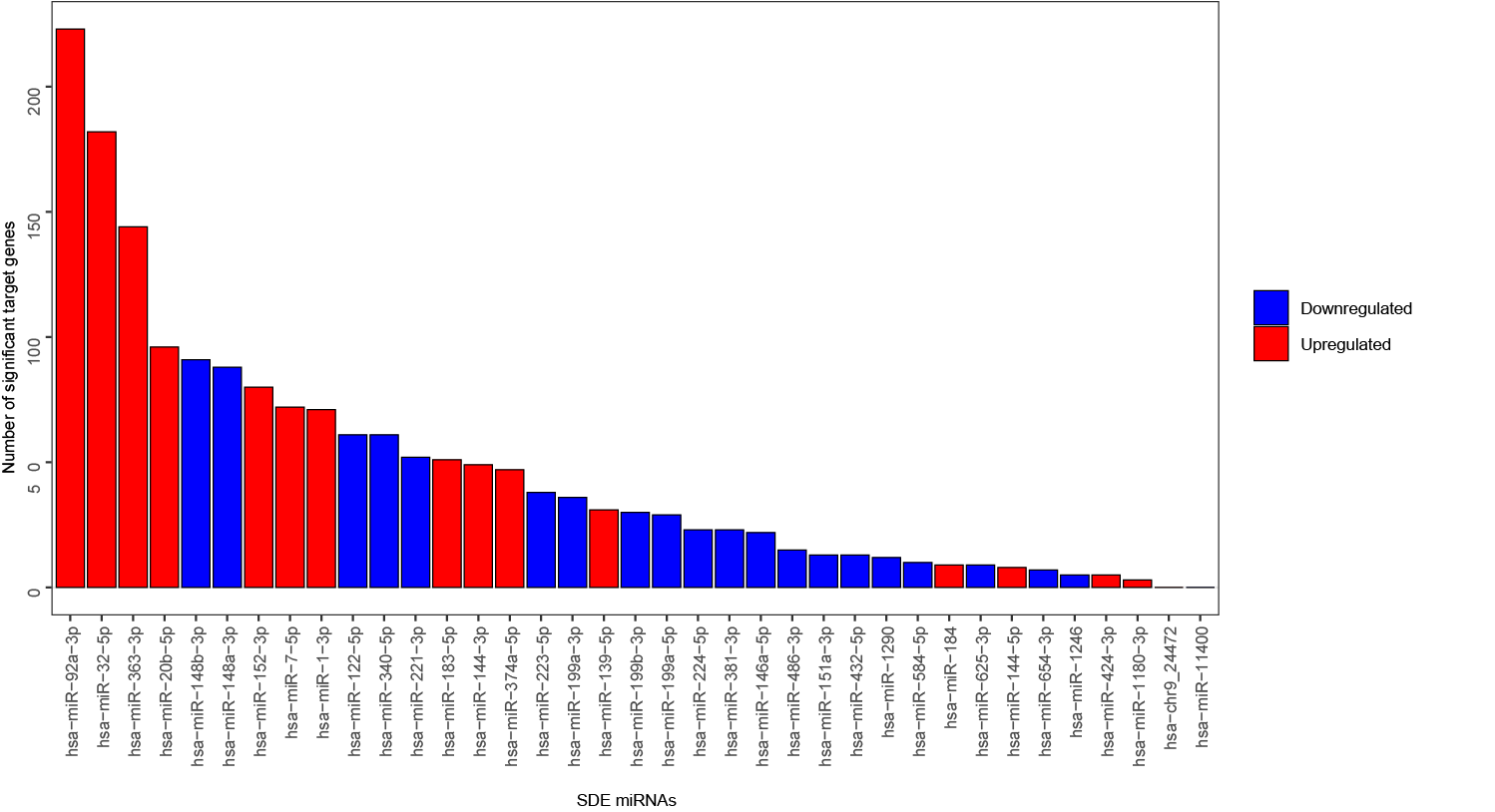
Upregulated miRNAs are represented in red and downregulated miRNAs in blue.

# **Supplementary Data 8.** Heatmap of pathway enrichment analysis.


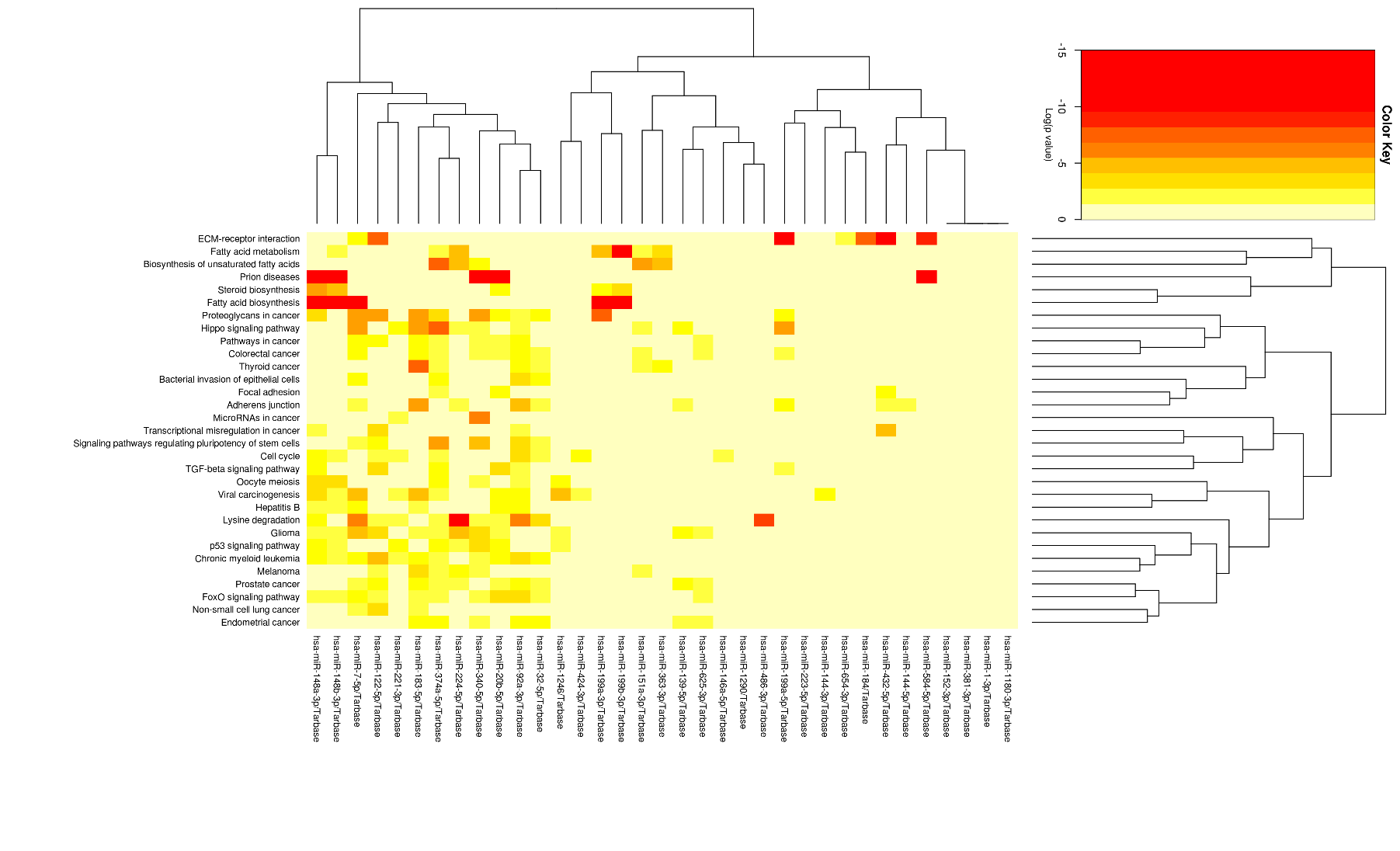
The significance of the association of SDE miRNAs with “Kyoto Encyclopedia of Genes and Genomes” categories is displayed in color (see color key), as identified applying the “pathways intersection” method.

# **Supplementary Data 9.** Top 25 pathways obtained in the pathway-enrichment analysis target genes of SDE miRNAs.

| **ID** | **Description** | **FDR** |
| --- | --- | --- |
| **KEGG** | | |
| **hsa05205** | Proteoglycans in cancer | 2.59E-14 |
| **hsa05206** | MicroRNAs in cancer | 6.41E-14 |
| **hsa05215** | Prostate cancer | 4.19E-13 |
| **hsa01522** | Endocrine resistance | 4.78E-12 |
| **hsa05210** | Colorectal cancer | 4.78E-12 |
| **hsa05165** | Human papillomavirus infection | 6.39E-12 |
| **hsa05224** | Breast cancer | 7.81E-12 |
| **hsa01521** | EGFR tyrosine kinase inhibitor resistance | 9.28E-12 |
| **hsa04151** | PI3K/AKT Signaling in Cancer | 2.74E-11 |
| **hsa05167** | Kaposi sarcoma-associated herpesvirus infection | 2.98E-11 |
| **hsa05226** | Gastric cancer | 6.07E-11 |
| **hsa05163** | Human cytomegalovirus infection | 9.29E-11 |
| **hsa05218** | Melanoma | 2.52E-10 |
| **hsa05225** | Hepatocellular carcinoma | 5E-10 |
| **hsa05212** | Pancreatic cancer | 5E-10 |
| **hsa05207** | Chemical carcinogenesis - receptor activation | 8.91E-10 |
| **hsa05161** | Hepatitis B | 1.61E-09 |
| **hsa05223** | Non-small cell lung cancer | 2.35E-09 |
| **hsa04933** | AGE-RAGE signaling pathway in diabetic complications | 2.35E-09 |
| **hsa04071** | Sphingolipid signaling pathway | 3.7E-09 |
| **hsa05214** | Glioma | 3.7E-09 |
| **hsa05160** | Hepatitis C | 5.32E-09 |
| **hsa04550** | Signaling pathways regulating pluripotency of stem cells | 7.87E-09 |
| **hsa04068** | FoxO signaling pathway | 1.43E-08 |
| **hsa04722** | Neurotrophin signaling pathway | 2.54E-08 |
| **REACTOME** | | |
| **R-HSA-5663202** | Diseases of signal transduction by growth factor receptors and second messengers | 4.8E-07 |
| **R-HSA-6785807** | Interleukin-4 and Interleukin-13 signaling | 4.8E-07 |
| **R-HSA-8848021** | Signaling by PTK6 | 3.41E-06 |
| **R-HSA-9006927** | Signaling by Non-Receptor Tyrosine Kinases | 3.41E-06 |
| **R-HSA-9634638** | Estrogen-dependent nuclear events downstream of ESR-membrane signaling | 8.83E-05 |
| **R-HSA-449147** | Signaling by Interleukins | 8.83E-05 |
| **R-HSA-9009391** | Extra-nuclear estrogen signaling | 8.83E-05 |
| **R-HSA-2219528** | PI3K/AKT Signaling in Cancer | 0.000181 |
| **R-HSA-9006925** | Intracellular signaling by second messengers | 0.000255 |
| **R-HSA-1257604** | PIP3 activates AKT signaling | 0.000354 |
| **R-HSA-2559585** | Oncogene Induced Senescence | 0.000428 |
| **R-HSA-8878166** | Transcriptional regulation by RUNX2 | 0.000815 |
| **R-HSA-109606** | Intrinsic Pathway for Apoptosis | 0.001093 |
| **R-HSA-199418** | Negative regulation of the PI3K/AKT network | 0.001465 |
| **R-HSA-8939211** | ESR-mediated signaling | 0.001465 |
| **R-HSA-166520** | Signaling by NTRKs | 0.001722 |
| **R-HSA-4420097** | VEGFA-VEGFR2 Pathway | 0.002711 |
| **R-HSA-3858494** | Beta-catenin independent WNT signaling | 0.00357 |
| **R-HSA-9006931** | Signaling by Nuclear Receptors | 0.003698 |
| **R-HSA-2559583** | Cellular Senescence | 0.003852 |
| **R-HSA-1227986** | Signaling by ERBB2 | 0.003852 |
| **R-HSA-5628897** | TP53 Regulates Metabolic Genes | 0.004158 |
| **R-HSA-194138** | Signaling by VEGF | 0.004211 |
| **R-HSA-3700989** | Transcriptional Regulation by TP53 | 0.004298 |
| **R-HSA-452723** | Transcriptional regulation of pluripotent stem cells | 0.004954 |
| **GO** | | |
| **GO:0036293** | response to decreased oxygen levels | 3.83E-09 |
| **GO:0001666** | response to hypoxia | 4.81E-09 |
| **GO:0070482** | response to oxygen levels | 6.75E-09 |
| **GO:0003158** | endothelium development | 1.14E-08 |
| **GO:0048732** | gland development | 1.36E-08 |
| **GO:0036294** | cellular response to decreased oxygen levels | 7.67E-08 |
| **GO:0071456** | cellular response to hypoxia | 1.9E-07 |
| **GO:0048608** | reproductive structure development | 1.9E-07 |
| **GO:0071453** | cellular response to oxygen levels | 1.9E-07 |
| **GO:0061458** | reproductive system development | 1.9E-07 |
| **GO:0061614** | pri-miRNA transcription by RNA polymerase II | 3.63E-07 |
| **GO:0045446** | endothelial cell differentiation | 3.63E-07 |
| **GO:0009791** | post-embryonic development | 5.67E-07 |
| **GO:1901653** | cellular response to peptide | 9.81E-07 |
| **GO:0018209** | peptidyl-serine modification | 2.69E-06 |
| **GO:0030900** | forebrain development | 2.69E-06 |
| **GO:0018105** | peptidyl-serine phosphorylation | 3.12E-06 |
| **GO:0010810** | regulation of cell-substrate adhesion | 6.05E-06 |
| **GO:0021543** | pallium development | 7.22E-06 |
| **GO:0002064** | epithelial cell development | 7.22E-06 |
| **GO:0006352** | DNA-templated transcription, initiation | 8.12E-06 |
| **GO:0050678** | regulation of epithelial cell proliferation | 1.03E-05 |
| **GO:0051893** | regulation of focal adhesion assembly | 1.49E-05 |
| **GO:0090109** | regulation of cell-substrate junction assembly | 1.49E-05 |
| **GO:0048569** | post-embryonic animal organ development | 1.49E-05 |

Each column represent: the pathway ID in each database format, the description of the pathway and the adjusted *p*-value (FDR, corrected p-value for multiple testing performed with the Benjamini–Hochberg procedure).

**References**

1. Kozomara, A. and S. Griffiths-Jones, *miRBase: annotating high confidence microRNAs using deep sequencing data.* Nucleic Acids Res, 2014. **42**(Database issue): p. D68-73.

2. Chen, Y., A.T. Lun, and G.K. Smyth, *From reads to genes to pathways: differential expression analysis of RNA-Seq experiments using Rsubread and the edgeR quasi-likelihood pipeline.* F1000Res, 2016. **5**: p. 1438.
